# Longitudinal Association of Built Environment Pattern with Physical Activity in a Community-Based Cohort of Elderly Hong Kong Chinese: A Latent Profile Analysis

**DOI:** 10.3390/ijerph17124275

**Published:** 2020-06-15

**Authors:** Jie-Sheng Lin, Faye Ya-Fen Chan, Jason Leung, Blanche Yu, Zhi-Hui Lu, Jean Woo, Timothy Kwok, Kevin Ka-Lun Lau

**Affiliations:** 1 Institute of Future Cities, The Chinese University of Hong Kong, Hong Kong 99077, China; yfchan@cuhk.edu.hk; 2 Jockey Club Centre for Osteoporosis Care and Control, The Chinese University of Hong Kong, Hong Kong 99077, China; jason-leung@cuhk.edu.hk (J.L.); blancheyu@cuhk.edu.hk (B.Y.); tkwok@cuhk.edu.hk (T.K.); 3 Department of Medicine and Therapeutics, Faculty of Medicine, The Chinese University of Hong Kong, Hong Kong 99077, China; anjolu@cuhk.edu.hk (Z.-H.L.); jeanwoowong@cuhk.edu.hk (J.W.); 4 CUHK Jockey Club Institute of Ageing, The Chinese University of Hong Kong, Hong Kong 99077, China

**Keywords:** built environment, built environment pattern, physical activity, latent profile analysis, cohort study

## Abstract

A large number of studies have focused on the associations between single built environment (BE) characteristics and physical activity (PA). Combinations of BE characteristics offer a more comprehensive approach to identify the BE–PA associations. We aimed to examine the BE–PA associations in a cohort of elderly Hong Kong Chinese. Between 2001 and 2003, 3944 participants (65–98 years of age) were recruited and followed for a mean of 7.8 years. BE characteristics were assessed via geographic information system. PA levels were obtained using the Physical Activity Scale for the Elderly questionnaire at baseline and three follow-ups. Latent profile analysis was first conducted to classify the BE characteristics, and linear mixed-effects models were then used to explore the longitudinal associations between the BE classes and changes in the PA levels. Three classes of BE were identified. Class 3 (characterized by greater green space and sky view factor) demonstrated a significant decline in household PA (β = −1.26, 95% confidence interval: −2.20, −0.33) during the study period, and a slower decline in walking PA (1.19 (0.42, 1.95)) compared with Class 2 (characterized by a greater proportion of residential land use). Our results indicate that BE patterns characterized by high green space and a sky view factor may help promote the walking PA level.

## 1. Introduction

Insufficient physical activity (PA) has become a global pandemic, with an estimated prevalence of 27.5% adults (≥18 years) with insufficient PA in 2016 globally, and 14.1% in China [1]. Older age groups (≥65 years) are more likely to have insufficient PA, with a prevalence ranging from 45.0 to 72.7% [2,3]. While previous studies highlighted the association of individual-level factors (e.g., socio-demographic characteristics and psychological factors) with PA [4], social ecological models which emphasize multi-level influences of individual, social and environmental factors have recently gained popularity in promoting health behaviors such as PA [5]. In particular, the built environment (BE), which refers to the human-made physical elements of living and working surroundings (e.g., buildings, infrastructures and green space), is one of the key factors in social ecological models [6]. 

The recognition of the role of BE in PA has grown. Green space, one of the most studied BE characteristics, was found to be positively associated with PA in general [7,8,9,10]. Other BE characteristics, such as residential density, public transport density and number of PA-related facilities, are also important to the promotion of PA [11,12,13,14,15,16]. While most previous studies have focused on the associations of individual BE characteristics with PA, a recent systematic review showed null consistent association of individual BE characteristics (e.g., accessibility to recreation and sidewalk coverage) with walking and cycling, but indices drawn from several BE characteristics (e.g., walkability index and density of land use) appeared to demonstrate consistent associations with walking and cycling in older adults [17]. The role of BE characteristics for the promotion of PA is complex, because different BE characteristics may have different impacts on PA that interact. Thus, combinations of BE characteristics offer a more comprehensive approach to identify the effect of BE on PA [18]. 

Data-driven statistical methods, such as k-means cluster analysis and supervised machine learning, are common approaches to identify patterns of complex BE characteristics. However, there are several limitations of these methods. For instance, the choice of cluster criteria of k-means cluster analysis is arbitrary, and supervised machine learning relies on a “training sample” with known a priori class memberships [19]. Latent profile analysis (LPA) can address the above limitations. LPA is a probability-based approach classifying participants into discrete clusters based on their distinctive response patterns, and allows for the statistical comparison of models to determine the number of clusters [19,20]. The main limitation of LPA is that, in some circumstances, redundant clusters may be identified, which means that the number of identified clusters may be more than the potential existing subgroups in the population [21].

Few studies have explored the associations of BE patterns derived by LPA using perceived or objective (obtained from geographic information system, GIS) BE characteristics with various types of PA in participants aged <65 years [22,23,24,25,26,27]. Among three studies of older adults over 65 years old [19,28,29], only one adopted objective BE characteristics with a small sample (*N* = 714) [19]. Todd et al. [19] used LPA with seven continuous GIS-measured BE characteristics to derive three representative BE classes, and found that the class characterized by high residential density, land use mix and recreation density had a higher PA level compared with the class characterized by low residential density, land use mix and intersection density in older adults. Another similar study, consisting of 11,541 adults from 11 countries, derived five BE classes from seven BE characteristics, and found that the proportion of participants grouped in each BE class varied by country (e.g., the proportion was higher for the U.S. in the “safe but activity unsupportive” class, while Hong Kong had a higher proportion in the “overall activity supportive” class), and the “overall activity supportive” class was more likely to meet the requirements of the PA guidelines compared with the “safe but activity unsupportive” class [27]. Such findings suggested that BE patterns differ across countries and have various impacts on PA. The above studies were mainly conducted in Western countries, while evidence from Asian countries is scarce. In addition, all the above studies were cross-sectional studies. Therefore, we aimed to derive BE patterns via LPA using GIS-based BE data and examined the longitudinal associations of the derived patterns with changes in PA level in a community-based cohort of elderly Chinese citizens in Hong Kong. 

This article includes five subsections: “Introduction”, “Materials and Methods”, “Results”, “Discussion” and “Conclusions”. In the Introduction, prior studies of the research field were reviewed. The limitations of prior studies and the main aim of this study were also highlighted. The Materials and Methods section describes the population with which this study was conducted and the data collected, including the means of collection, and provides detailed information on statistical analyses. A precise description of the research findings is provided in the Results section. In the Discussion section, we discuss the results and how they can be explained based on previous research. Finally, the main findings and their implications are summarized in the Conclusions section. The novelties of this study include the use of LPA with GIS-based data and the use of a large longitudinal sample.

## 2. Materials and Methods 

### 2.1. Study Design and Population 

This study was based on the Mr. OS and Ms. OS Study (Hong Kong), a cohort study designed to examine the nutritional determinants and lifestyle factors (e.g., PA and alcohol drinking) of bone health, body composition and other health outcomes in older Chinese men and women [30,31,32]. Almost 200 publications have been published based on this cohort in more than 20 areas, including fractures and body fat. Previous studies based on this cohort have focused on the associations of individual and social factors with various health outcomes, while social ecological models have highlighted the importance of BE in promoting health. Few studies based on this cohort have shown favorable associations of BE characteristics (e.g., green space) with frailty and mortality [33,34]. We hypothesized that BE may reduce the risk of mortality by mediating PA. Therefore, we aimed to examine the longitudinal associations of BE with PA level in this cohort.

Between 2001 and 2003, 2000 men and 2000 women aged 65–98 years living in the community were recruited via public advertisement at community centers in Hong Kong. Those who were unable to walk independently, had a bilateral hip replacement, and were not competent to give informed consent were excluded. A stratified sampling method was used to ensure the even distribution of age; approximately 33% participants were in each of the age groups: 65–69, 70–74, and ≥75 years. Compared with the general older adults aged 65 years and older in Hong Kong, participants in our cohort were more likely to be married (70.9% vs. 59.9%), less likely to be live alone (10.7% vs. 11.3%) and had a higher education level (9.8% vs. 3.8% with higher education) [35]. Details of the study population have been reported elsewhere [30,31,32].

**Figure 1:**
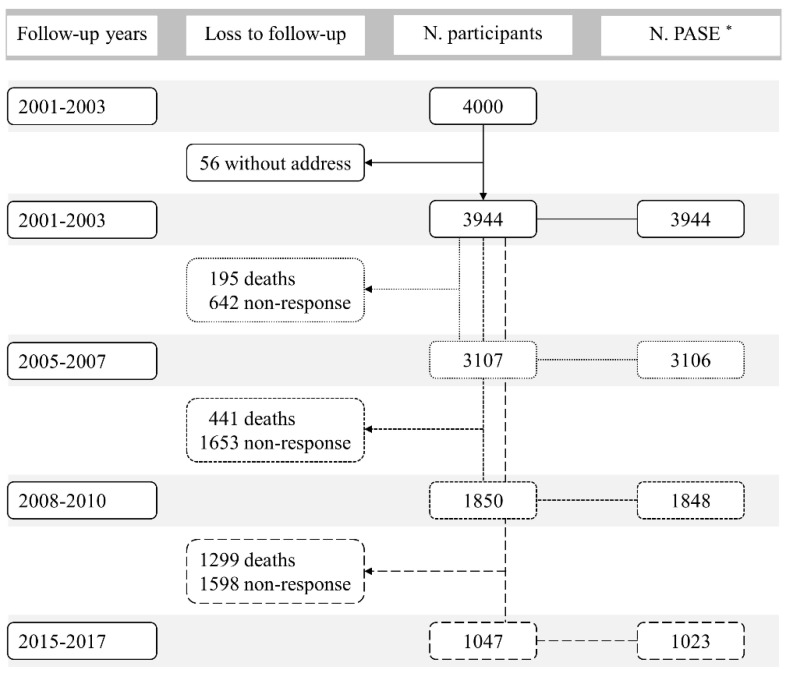
Flowchart of the study participants over the study period. * Number of participants who completed the Physical Activity Scale for the Elderly (PASE).

The participants were followed during the periods 2003–2005 (without assessing PA), 2005–2007, 2008–2010 and 2015–2017, i.e., four times. Participants without a valid address (*n* = 56) at baseline, and missing all PA assessments were excluded from the present study. The flow chart of participants included in this study is presented in Figure 1. Finally, 3944 participants with a total of 9921 observations were included in the present analyses. The mean follow-up time was 7.8 years. The study was approved by the Clinical Research Ethics Committee of the Chinese University of Hong Kong, and all participants provided written informed consent.

### 2.2. Measurement of Built Environment 

GIS-based BE data in the format of shapefiles including building height, ground coverage of the building, green spaces and land use types, were obtained from the iB1000 Topographical Dataset of the Hong Kong Lands Department, and subsequently rasterized with a spatial resolution of 1 m, using ArcGIS 10.3 software (ESRI Inc. Redlands, CA, USA). This dataset was obtained from the government records of buildings and their master layout plan, as well as land use zoning information. The dataset is regularly updated and the data updated in 2018 were used in this study. The sky view factor (SVF), a morphological parameter describing the openness of a specific point location, was calculated using Equation (1) [36]. The normalized difference vegetation index was calculated from IKONOS multispectral images with a spatial resolution of 15 m in order to represent the live vegetation, i.e., green space in urban areas [37]. A 300 m Euclidean buffer around the participants’ addresses (around a five-minute walking distance, as our previous study reported an average walking speed of 1.0 m/s in our participants [38]), was used to calculate the standardized building height, the percentage of building ground coverage, green spaces, and the percentage of industrial, residential, commercial, government, institution and community land uses (Table 1) to represent the neighborhood environment that the participants were exposed to:

(1) $$SVF=\frac{1}{2\pi }\overset{2\pi }{\underset{0}{\int }}\left[\cos\beta \;\cos^{2}\varphi +\sin\beta \cdot \cos\left(\Phi -\alpha \right)\cdot \left(90-\varphi -\sin\varphi \cos\varphi \right)\right]d\Phi$$

### 2.3. Self-Reported Physical Activity

The PA level was assessed using the validated Physical Activity Scale for the Elderly (PASE) questionnaire [39,40,41] at baseline 2001–2003, and follow-ups in the years of 2005–2007, 2008–2010 and 2015–2017. The PASE is a widely used measure in epidemiological studies to assess the PA level of older adults, and has been found to be a reliable and valid tool for assessing the PA level of the elderly in the Chinese population [42,43]. The PASE comprised 12 self-reported items over a one-week period, including leisure PA (including walking outside home, light/moderate/strenuous sport activity and muscle strength exercise), household PA (including light/heavy housework, home repairs, lawn work or yard care, outdoor gardening and caring for another person) and occupational PA (work for pay or as a volunteer). Participants first reported how often (i.e., never, 0 day; seldom, 1–2 days; sometimes, 3–4 days; or often, 5–7 days) per week, and then estimated the number of hours per day (i.e., less than 1 h, 1–2 h, 2–4 h, or more than 4 h) a variety of activities were performed. PASE scores were calculated by multiplying the empirically derived activity weights and frequency values of each of the 12 types of activities. A summary score was calculated to represent the daily total PA level. The questionnaire was adapted in Hong Kong by adding activity items that are popular in the local culture, such as Mahjong and Tai Chi. We monitored the PA of a subgroup participants (363 men and 287 women) by Actigraph accelerometer in our cohort and found that the total PASE score was significantly correlated with the total activity counts per day (r = 0.33 for man and 0.43 for women). 

### 2.4. Covariates

A questionnaire was administered by trained investigators in face-to-face interviews to collect the following information: (1) socio-demographic characteristics: age, sex, marital status (married/widowed/separated or divorced/never married) and education level (no education/primary school or below/secondary school or above); (2) lifestyles: alcohol drinking (Have you been drinking alcohol in the past year? yes/no) and smoking (Have you been smoking in the past year? yes/no); (3) living condition: years lived in Hong Kong and living alone (yes/no); (4) health conditions of self-rated health (very poor/poor/fair/good/very good) and the history of chronic diseases and medications; and (5) general mental health and cognitive function. Instrumental activities of daily living was used to measure the functional impairment (number of activities participants cannot do). The number of chronic diseases was calculated through the self-reported presence of the following chronic diseases or symptoms: diabetes, hypertension, cardiovascular diseases, stroke, cancer, Parkinson’s disease, chronic obstructive lung disease, glaucoma, cataracts, arthritis and dizziness. It was then categorized into 0, 1–2 or ≥3 chronic diseases. The Chinese Geriatric Depression Scale was used to detect depression (cutoff ≥ 8). The Mini-Mental State Examination (MMSE) and Community Screening Interview for Dementia (CSI-D) were used to estimate cognitive function.

### 2.5. Statistical Analyses 

#### 2.5.1. Latent Profile Analysis

To reduce the dimensionality of 10 BE characteristics (continuous variables), the LPA was used to derive several discrete classes (Table 1 and Table S1). To determine the optimal number of classes for our data, models with 1–9 classes were identified, and the model selection was determined by a series of model fit statistics, such as Akaike information criteria (AIC), Bayesian information criteria (BIC), sample-size-adjusted BIC (SSA-BIC), bootstrap likelihood ratio test (BLRT), entropy ≥ 0.9, interpretability of the classes and the size of class membership [20].

#### 2.5.2. Inverse Probability Weighting

To address bias caused by the differential loss to follow-up, the inverse probability weighting method [44] was used to examine the impact of differential loss to follow-up in the present study. Reasons of loss to follow-up were divided into death and other causes (e.g., refusal and inability to contact). The probability of death (yes/no) and loss to follow-up due to other causes (yes/no) were estimated for each observation using a generalized estimating equation with relevant baseline time-constant covariates (e.g., sex and education level) and time-varying covariates as predictors (e.g., alcohol drinking and smoking). The weights of some observations may potentially be very large, and thus we further calculated the stabilized probability weights. Detailed information is provided in the Supplementary Information (Text S1). Of the total 9921 observations, seven values were missing for depression, 11 values for functional impairment, 198 values for alcohol drinking and 353 values for smoking. Thus, multiple imputation using chained equations was used to replace the missing data in these covariates.

#### 2.5.3. Main Analyses

ANOVA (for continuous variables) or chi-squared test (for categorical variables) was used to determine whether there were significant differences in the baseline characteristics of the study participants between the three BE classes. To take into account repeated measurements for each participant, weighted linear mixed-effects models were used to explore the associations between the BE class and change in the level of PA, using age (centered at mean age at baseline, 72.5 years, and divided by 5 to give change over 5 years) as a timescale. The fixed effect included BE class, age, and their interaction term. As random effects, we used intercepts for participants (random intercept), as well as by-participants slopes for the effect of change in PA (random slope). The estimated effect of the interaction term between the BE class and age reflected the impact of the BE class on annual change in PA (longitudinal association). The main effect of BE class reflected the baseline difference in PA that were associated with the BE class (cross-sectional association). Since previous studies suggested that certain covariates might affect the associations of BE with PA [19,23,24,26,29,34,45], such as diluting the associations, three models were constructed to control potential confounders: Model 1—adjusted for sex; Model 2—adjusted for sex, marital status, education level, alcohol drinking, smoking, years lived in Hong Kong, living alone, self-rated health, depression, MMSE, CSI-D and number of chronic diseases; covariates in Model 2 were treated as time-varying variables, except sex, education level, years lived in Hong Kong and CSI-D; Model 3—adjusted for all covariates in Model 2 plus baseline PA level (total, leisure, household and walking PA, respectively) to minimize the possibility of reverse causality. 

#### 2.5.4. Sensitivity Analyses

Several sensitivity analyses were performed based on Model 3 to test the robustness of our findings. We repeated the main analyses (a) using unweighted linear mixed-effects models, (b) excluding participants who reported moving from the baseline address during follow-up, (c) excluding participants who were lost to follow-up within 4 years after baseline, (d) excluding observations in the 2015–2017 follow-up, and (e) excluding participants who reported a functional impairment (number of activities participants cannot do ≥1).

All analyses were conducted by R version 3.6.1 (R Development Core Team, Vienna, Austria) and RStudio version 1.2.1335 (RStudio, Boston, MA, USA), using “tidyLPA”, “geepack”, “lme4” packages. A two-tailed *p*-value < 0.05 was considered statistically significant.

## 3. Results

### 3.1. Latent Profile Analysis 

**Figure 2:**
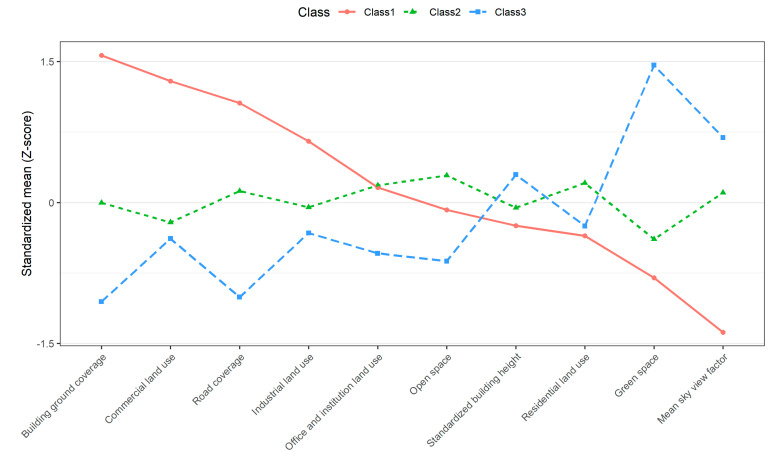
Standardized mean (Z-score) for each built environment characteristic by latent class.

The tests of the class solutions are described in Table S1. Models of 4–9 classes had a smaller entropy value (≤0.89), a minimum of the average class diagonal probabilities (≤89%), and contributed small classes with about 1–5% of the sample (Table S1). Comparisons between three-class and four-class models revealed that the three-class model fitted the data better (entropy = 0.9, minimum of the average class diagonal probabilities = 93%, and contributed small classes with 17%). Figure 2 shows the standardized mean (Z-score) for each BE characteristic, by the three-class model and Figure S1 shows the geographical distribution of the participants’ addresses at baseline. Class 1 was labeled “commercial area”, characterized by high building ground coverage, commercial land use, road coverage and industrial land use, and represented 16.9% of the sample (*N* = 665). In contrast, Class 3 was characterized by a high coverage of green space and mean SVF, which accounted for 24.8% of the sample (*N* = 979) and was named “area near green space”. Class 2 (“residential area”) was the largest class (59.3%, *N* = 2300), characterized by a high coverage of open space, residential land use and a mix of other BE characteristics.

### 3.2. Inverse Probability Weighting 

In these multivariable-adjusted analyses, the age and number of chronic diseases were strong predictors of death and loss in follow-up due to other causes. In contrast, a higher CSI-D score and total PA level at baseline were associated with a lower risk of death and loss in follow-up due to other causes. Other predictors of death included being male, widowed or not married, longer years lived in Hong Kong, smoking, worse self-rated health and lower MMSE score. Those with a higher education level, longer years lived in Hong Kong and had depression were less likely to drop out (Table S2).

### 3.3. Main Analyses

The characteristics of participants over the study period are presented in Table S3. The median total PA score was 84.8 in 2001–2003, 95.6 in 2005–2007, 99.1 in 2008–2010 and declined to 68.4 in 2015–2017. Table 2 presents the baseline characteristics by the three-class model. Participants in Class 3 (“area near green space”) were more likely to be older, female, be separated/divorced/single, and have a lower education level, MMSE score and CIS-D score.

**Figure 3:**
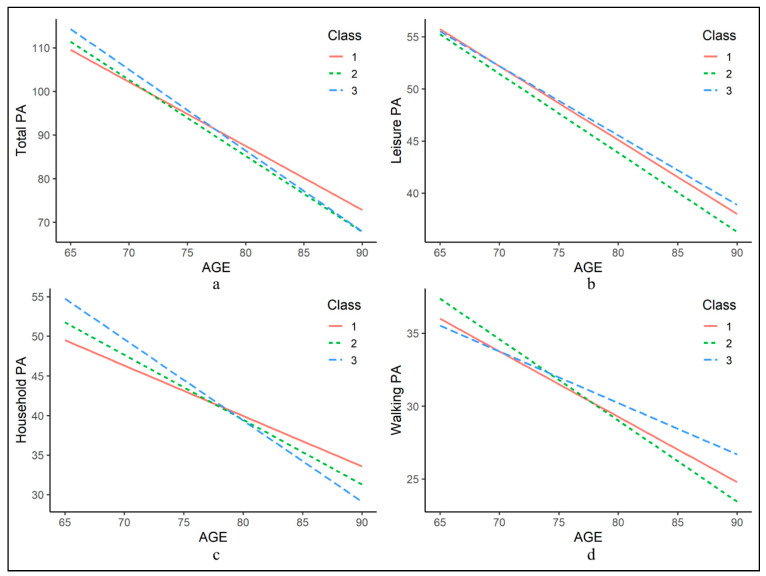
Trajectories of physical activity (PA) over age by built environment class. Trajectories derived from linear mixed-effects models, adjusted for age, sex, marital status, education level, alcohol drinking, smoking, years lived in Hong Kong, living alone, self-rated health, depression, Mini-Mental State, Examination Community Screening Interview for Dementia, number of chronic diseases and level of baseline total, leisure, household and walking physical activity.

A series of weighted linear mixed-effects analyses was conducted to explore the associations between the BE classes and changes of PA levels over time (Table 3 and Figure 3). Overall, there were no associations of BE class with changes in the level of total and leisure PA across Models 1, 2 and 3. After adjustment for potential confounders (Model 2), Class 3 had a faster household PA decline over 5 years (β = −1.39, 95% confidence interval (CI): −2.74, −0.04) compared with Class 1 and Class 2 (β = −1.17, 95% CI: −2.20, −0.14). With further adjustment for baseline household PA (Model 3), no significant differences were observed between Class 3 and Class 1, but the difference between Class 3 and Class 2 remained significant (β = −1.26, 95% CI: −2.20, −0.33). On the contrary, Class 3 had a slower decline in walking PA in comparison with Class 2 over the three models, with β = 1.19 (95% CI: 0.42, 1.95) for Model 3. The baseline difference was only found in household PA, and Class 3 had a higher level of baseline household PA than Class 1, with (β = 2.20, 95% CI: 0.10, 4.29) for Model 2. There were no substantial differences in the various sensitivity analyses (Table S4).

## 4. Discussion

In this prospective cohort study of elderly Hong Kong Chinese men and women, we classified 10 GIS-based BE characteristics into three BE classes by LPA, and investigated the longitudinal associations between the BE class and the decline in the level of PA. We found that Class 3 (“area near green space”) had a faster household PA decline and a slower decline in walking PA compared with Class 2 (“residential area”).

One of the main findings in the present study was that those who lived near more green space and with a higher SVF (Class 3) had a slower decline in walking PA compared with participants living in residential areas (Class 2, characterized by high open space and residential land use). It was against our expectations that those who lived in a residential area had a faster decline in walking PA, since prior cross-sectional studies have reported that LPA-derived BE classes, characterized by high residential density and park access, were positively associated with transport-related walking PA in adults [23,24,29]. The inconsistent findings between prior studies and this study are likely due to different combinations of BE characteristics. In addition to high residential density and park access, BE classes associated with higher transport-related walking PA in prior studies were characterized by high-land use mix, recreation facilities access and walking facilities [23,24,29]. Different study designs partially explain the inconsistencies. Similar to prior studies, our results also suggested that Class 2 tended to have a higher walking PA at baseline (non-significant), but this was inconsistent with the results from the longitudinal analysis.

On the other hand, Class 3 had greater proportion of green space compared with Class 2. People living near green space are more likely to have a better chance for walking, and previous studies highlighted that a higher level of green space was associated with a slow decline in recreational walking PA [7,8,9], which was consistent with our findings. However, a cross-sectional study observed that green space was inversely associated with active transport (walking or cycling to a destination) in Canadian adults aged 20–89 years [46]. One possible reason for the inconsistent results is that active transport users are more likely to travel through less green space areas in order to reach their destination faster. In addition, Class 3 was also characterized by high SVF, which is a less-studied BE characteristic in health studies, and there has been no previous study regarding the association of SVF with PA. It was suggested that a 10% increase in SVF led to a 7–8% increase in wind speed and improved thermal comfort at the pedestrian level [47], which encourages walking PA. A pedestrian-friendly wind environment should therefore be considered in urban design. On the contrary, a higher value of SVF, leading to a higher daytime air temperature and a lower level of shading, was related to a lower level of thermal comfort in summer [48], and therefore might reduce the probability of walking outside in summer. However, walking in the early morning (5–10 a.m.) was reported to be the most popular PA in Hong Kong older adults [49,50], and our previous study, which monitored the PA of a subgroup participants in our cohort (*N* = 641) by Actigraph accelerometer, also found that PA peaked in the morning [51]. In summer, thermal comfort conditions in the morning are better than those at noon and in the afternoon. Thus, the overall lower level of thermal comfort on summer days might not have a significant negative impact on walking PA among our participants. The above evidence supports our finding that participants living near higher levels of green space and with a higher SVF had a slower decline in walking PA.

Inexplicably, we also found that Class 2 had a slower household PA decline compared with Class 3. It was possible that people living in residential areas and with less green space had fewer chances for outdoor activities so they spent more time on household PA. Thus, a higher proportion of time spent in household PA might reflect a lower proportion of time spent in other domains of PA, such as walking PA and leisure PA. BE might not have a direct impact on household PA but have a direct impact on other domains of PA, so the association of BE with household PA we observed was likely an indirect association. An accidental finding by chance was also possible since, in sensitivity analyses, the associations were not significant after excluding observations in the 2015–2017 follow-up or excluding participants who reported a functional impairment. Previous studies reported that household PA was largely determined by sex, age and social economic status [4], but we found no previous studies that focused on BE. Therefore, further investigations are needed.

This study has several strengths. First, to the best of our knowledge, the present study is the first longitudinal study to investigate the association of objective BE pattern derived by LPA with PA change in older adults. Second, a variety of time-varying and time-constant covariates were included in our statistical models, and several sensitivity analyses were conducted, which supported our findings.

The following limitations should also be considered. First, GIS-based BE characteristics were only measured at baseline and they might have changed during follow-up (the neighborhood BE changed or participants moved from the baseline address), which might have diluted the association. However, the results of the sensitivity analyses were robust after excluding participants who reported moving from the baseline address during follow-up and previous evidence indicated that BE characteristics, such as land use, did not change substantially within a decade [52]. Second, PA was measured based on self-reported data and the correlation with accelerometer-measured PA was relatively low (r = 0.33 for man and 0.43 for women), which might have been subject to error and weakened the magnitude of associations between BE and walking PA. Third, participants were lost in the follow-up due to death or other reasons in each follow-up, which may have led to selection bias. However, we used the inverse probability weighting method [44] to address this limitation. Fourth, using LPA-derived BE classes might have made it difficult to identify where the impact of PA was actually coming from. Fifth, although multiple potential confounders were controlled for, residual confounding due to unmeasured confounders could not be excluded, which might have diluted the association. Finally, the present study was based on older adults aged 65 years and older in Hong Kong, an ultra-dense Chinese metropolis, and thus, the generalization of our findings should be undertaken with caution.

## 5. Conclusions

In this community-based cohort of elderly Hong Kong Chinese, we found that people living in neighborhoods characterized by high green space and SVF had a slower decline of walking PA, since these people are likely to have better opportunities for walking. No significant difference was observed for the total PA level. Our results add to the literature that indicates a certain pattern of BE characteristics, such as a BE pattern characterized by a high green space and SVF, which may help promote walking PA level in older adults. Our findings provide urban planners and public health policy makers with evidence of the potential benefits of certain BE patterns for walking PA, and indicate that urban design promoting a walking PA level should consider combinations of multiple BE characteristics. More investigations are warranted to replicate our findings, especially for longitudinal studies.

## Figures and Tables

**Table 1:** Built environment characteristics.

	Median	Interquartile Range	Min	Max
Standardized building height, m	31.8	14.1	0.00	74.8
Mean sky view factor	0.62	0.15	0.25	1.00
Building ground coverage, %	21.0	14.0	0.00	52.0
Green space, %	13.1	29.5	0.00	100
Open space, %	5.06	8.14	0.00	47.9
Road coverage, %	17.2	8.56	0.00	37.9
Industrial land use, %	0.00	1.03	0.00	38.3
Residential land use, %	36.9	17.0	0.00	99.5
Commercial land use, %	0.07	1.45	0.00	32.4
Government, institution and community land use, %	6.54	7.43	0.00	51.7

**Table 2:** Baseline characteristics of the participants by built environment class.

	Class 1 (*N* = 665)	Class 2 (*N* = 2300)	Class 3 (*N* = 979)	*p*-Value ^1^
	Mean (SD) or Number (%)			
Age, years	71.9 (4.84)	72.6 (5.25)	72.7 (5.26)	0.004
Sex, female, *N* (%)	273 (41.1)	1182 (51.4)	517 (52.8)	<0.001
Marital status, *N* (%)				<0.001
Married	525 (78.9)	1596 (69.4)	674 (68.8)	
Widowed	117 (17.6)	603 (26.2)	251 (25.6)	
Separated or divorced	10 (1.5)	51 (2.2)	27 (2.8)	
Single (never married)	13 (2.0)	50 (2.2)	27 (2.8)	
Education level, *N* (%)				<0.001
No education	90 (13.5)	525 (22.8)	228 (23.3)	
Primary school or below	308 (46.3)	1137 (49.4)	532 (54.3)	
Secondary school or above	267 (40.2)	638 (27.8)	219 (22.4)	
Years lived in Hong Kong	53.0 (14.7)	53.0 (14.7)	52.1 (15.5)	0.216
Alcohol drinking, *N* (%)	102 (15.3)	295 (12.8)	118 (12.1)	0.137
Smoking, *N* (%)	43 (6.5)	157 (6.8)	73 (7.5)	0.711
Number of chronic diseases, *N* (%)				0.030
0	117 (17.6)	390 (17.0)	141 (14.4)	
1 or 2	376 (56.5)	1230 (53.5)	576 (58.8)	
≥3	172 (25.9)	680 (29.5)	262 (26.8)	
Self-rated health, *N* (%)				0.151
Very poor, poor or fair	329 (49.5)	1223 (53.2)	530 (54.1)	
Good or very good	336 (50.5)	1077 (46.8)	449 (45.9)	
Live alone, *N* (%)	61 (9.2)	270 (11.7)	92 (9.4)	0.054
Depression, *N* (%)	45 (6.8)	224 (9.7)	96 (9.8)	0.052
MMSE	26.3 (3.19)	25.5 (3.78)	25.5 (3.71)	<0.001
CSI-D	30.6 (1.65)	30.1 (2.08)	30.0 (2.13)	<0.001

Abbreviations: CSI-D, Community Screening Interview for Dementia; MMSE, Mini-Mental State Examination; SD, standard deviation. ^1^ *p*-value was estimated by ANOVA (continuous variables) or chi-squared test (categorical variables).

**Table 3:** Difference in the level of physical activity (PA) at baseline and change over 5 years with built environment class (case = 3944; observation = 9921) ^1^.

	Model 1 ^2^		Model 2 ^3^		Model 3 ^4^
	Baseline	5-Year Change	Baseline	5-Year Change	5-Year Change
Total PA					
Class 2 vs. 1	0.07 (−3.05, 3.18)	−0.83 (−2.89, 1.22)	0.22 (−2.85, 3.29)	−0.87 (−2.85, 1.12)	0.16 (−1.66, 1.99)
Class 3 vs. 1	2.25 (−1.34, 5.83)	−1.76 (−4.13, 0.60)	2.37 (−1.18, 5.91)	−1.23 (−3.51, 1.05)	−0.17 (−2.27, 1.92)
Class 3 vs. 2	2.18 (−0.59, 4.95)	−0.93 (−2.73, 0.87)	2.14 (−0.58, 4.87)	−0.36 (−2.10, 1.37)	−0.34 (−1.93, 1.25)
Leisure PA					
Class 2 vs. 1	−0.33 (−2.23, 1.57)	−0.41 (−1.69, 0.87)	−0.34 (−2.21, 1.54)	−0.39 (−1.65, 0.86)	−0.26 (−1.43, 0.91)
Class 3 vs. 1	0.64 (−1.54, 2.83)	0.02 (−1.45, 1.49)	0.60 (−1.57, 2.76)	0.30 (−1.13, 1.74)	0.68 (−0.66, 2.03)
Class 3 vs. 2	0.97 (−0.72, 2.66)	0.43 (−0.69, 1.55)	0.93 (−0.73, 2.60)	0.70 (−0.40, 1.79)	0.94 (−0.08, 1.97)
Household PA					
Class 2 vs. 1	0.54 (−1.29, 2.36)	−0.20 (−1.41, 1.00)	0.69 (−1.12, 2.51)	−0.22 (−1.39, 0.95)	0.44 (−0.64, 1.51)
Class 3 vs. 1	2.05 (−0.05, 4.15)	−1.69 (−3.08, −0.31) ^*^	2.20 (0.10, 4.29) ^*^	−1.39 (−2.74, −0.04) ^*^	−0.83 (−2.06, 0.40)
Class 3 vs. 2	1.52 (−0.10, 3.14)	−1.49 (−2.55, −0.44) ^**^	1.50 (−0.10, 3.11)	−1.17 (−2.20, −0.14) ^*^	−1.26 (−2.20, −0.33) ^**^
Walking PA					
Class 2 vs. 1	1.05 (−0.32, 2.41)	−0.60 (−1.54, 0.35)	0.91 (−0.44, 2.27)	−0.58 (−1.51, 0.36)	−0.46 (−1.33, 0.42)
Class 3 vs. 1	0.77 (−0.80, 2.34)	0.41 (−0.67, 1.50)	0.58 (−0.98, 2.15)	0.56 (−0.52, 1.63)	0.73 (−0.27, 1.73)
Class 3 vs. 2	−0.28 (−1.49, 0.93)	1.01 (0.18, 1.83) ^*^	−0.33 (−1.54, 0.87)	1.13 (0.31, 1.95) ^**^	1.19 (0.42, 1.95) ^**^

^1^ β (95% confidence interval) were estimated from weighted linear mixed-effects models. ^2^ Model 1: including age, built environment class and their interaction term (age × built environment class), and adjusted for sex. ^3^ Model 2: Model 1 plus marital status, education level, alcohol drinking, smoking, years lived in Hong Kong, living alone, self-rated health, depression, Mini-Mental State Examination, Community Screening Interview for Dementia and number of chronic diseases. ^4^ Model 3: Model 2 plus level of baseline total, leisure, household and walking physical activity, respectively. * *p*-value < 0.05, ** *p*-value < 0.01, *** *p*-value < 0.001.

## Supplementary Materials

The following are available online at https://www.mdpi.com/1660-4601/17/12/4275/s1, Text S1. Additional information on the inverse probability weighting analysis; Table S1. Model fit statistics for latent profile analysis of 1 to 9 class models and class probabilities; Table S2. Adjusted HR (hazard ratio) and 95%CI (confidence interval) of attrition due to loss to follow-up over the study period; Table S3. Characteristics of participants and the level of physical activity over the study period; Table S4. Sensitivity analyses for difference in the level of physical activity (PA) change over 5 years with built environment class; Figure S1. Geographical distribution of the participants’ addresses at baseline.
