# Supplementary material for: Longitudinal Association of Built Environment Pattern with Physical Activity in a Community-Based Cohort of Elderly Hong Kong Chinese: A Latent Profile Analysis
Source: Int J Environ Res Public Health. 2020 Jun 15;17(12):4275. doi: 10.3390/ijerph17124275 (PMC7344458; doi:10.3390/ijerph17124275)
Supplement: Supplementary file 1 [file ijerph-17-04275-s001.pdf]

Online supplemental materials

Longitudinal association of built environment pattern with physical activity in a community-based cohort of elderly Hong Kong Chinese: a latent profile analysis

Jie-Sheng Lin, Faye Ya-Fen Chan, Jason Leung, Blanche Yu, Zhi-Hui Lu, Jean Woo, Timothy Kwok, Kevin Ka-Lun Lau

Text S1. Additional information on the inverse probability weighting analysis

Methods

For addressing bias caused by differential loss to follow-up, inverse probability weighting method [1] was used to examine the impact of differential loss to follow-up in the present study. The reasons of loss to follow-up were divided into death and other causes (e.g., refusal and inability to contact), and because determinants of death and loss to follow-up for other causes may be different, we separately modeled attrition due to death and attrition due to other causes.

First, each observation`s probability of death (P1) and loss to follow-up due to other causes (P2) in each wave of follow-up were estimated by generalized estimating equation (GEE) regression models with death (yes / no) and loss to follow-up due to other causes (yes / no) as outcomes, including baseline time-constant covariates (sex, education level, years lived in Hong Kong and Community Screening Interview for Dementia), time-varying covariates (age, marital status, alcohol drinking, smoking, living alone, self-rated health, depression, Mini-Mental State Examination and number of chronic diseases), level of baseline total physical activity and built environment class as predictors. Non-stabilized inverse probability weighting was calculated as 1 / (P1*P2). As the reciprocal of a probability, some observation`s probability may potentially be very large for participants with a small probability of staying alive and being in follow-up. We further calculated stabilized weights.

Second, each observation`s probability of death (S1) and loss to follow-up due to other causes (S2) in the study were estimated by GEE regression models with death (yes / no) and loss to follow-up due to other causes (yes / no) as outcomes, including sex, education level, years lived in Hong Kong, Community Screening Interview for Dementia, marital status, alcohol drinking, smoking, living alone, self-rated health, depression, Mini-Mental State Examination and number of chronic diseases (only baseline time-constant covariates) as predictors. Stabilized weights were calculated as (S1*S2) / (P1*P2), and used in the main analyses.

Reference

1. Weuve, J.; Tchetgen, E.J.T.; Glymour, M.M.; Beck, T.L.; Aggarwal, N.T.; Wilson, R.S.; Evans, D.A.; de Leon, C.F.M. Accounting for bias due to selective attrition the example of smoking and cognitive decline. *Epidemiology* **2012**, *23*, 119-128.

Table S1. Model fit statistics for latent profile analysis of 1 to 9 class models and class probabilities

Fit statistics	1 Class	2 Class	3 Class	4 Class	5 Class	6 Class	7 Class	8 Class	9 Class
Log-likelihood	-101204.08	-98051.94	-95832.30	-94471.85	-93814.94	-92525.07	-92337.04	-91398.31	-90689.96
AIC	202448.17	196165.88	191748.61	189049.70	187757.87	185200.15	184846.08	182990.61	181595.93
BIC	202573.77	196360.56	192012.37	189382.53	188159.79	185671.14	185386.16	183599.77	182274.16
SSA-BIC	202510.21	196262.05	191878.91	189214.12	187956.43	185432.83	185112.89	183291.54	181930.99
Entropy		0.91	0.90	0.89	0.85	0.87	0.88	0.87	0.89
BLRT		6304.00	4439.38	1452.89	1731.06	2441.57	2542.62	779.31	1333.03
BLRT p-value		< 0.001	< 0.001	< 0.001	< 0.001	< 0.001	< 0.001	< 0.001	< 0.001
Prob. min		0.95	0.93	0.89	0.84	0.87	0.86	0.88	0.83
Prob. max		0.98	0.96	0.96	0.97	0.97	0.96	0.98	0.97
N. min		0.28	0.17	0.04	0.04	0.05	0.03	0.01	0.03
N. max		0.72	0.58	0.53	0.36	0.29	0.32	0.28	0.30

AIC, Akaike Information Criterion; BIC, Bayesian Information Criterion; SSA-BIC, Sample-size-adjusted BIC: lower values on these fit statistics indicate better model fit.
BLRT, Bootstrap Likelihood Ratio Test: comparing the improvement between neighboring class models.
Entropy: a measure of the precision of classification, higher value indicates greater precision.
Prob. min / max: minimum / maximum of the average latent class probabilities for most likely class membership by assigned class.
N. min / max: proportion of the sample assigned to the smallest / largest class (based on most likely class membership).

Table S2. Adjusted HR (hazard ratio) and 95%CI (confidence interval) of attrition due to loss to follow-up over the study period [a]

	Adjusted HR (95% CI)	
	Death (case = 3944; observation = 9921)	Other causes (case = 3466; observation = 8622)
Age, per SD, prior	1.41 (1.34, 1.49) ***	1.16 (1.11, 1.21) ***
Female (ref: male)	0.37 (0.32, 0.42) ***	1.08 (0.97, 1.20)
Marital status, prior (ref: married)		
Widowed	1.20 (1.04, 1.38) *	0.95 (0.85, 1.08)
Separated or divorced	1.31 (0.94, 1.82)	0.91 (0.67, 1.24)
Single (never married)	2.17 (1.59, 2.95) ***	0.99 (0.70, 1.41)
Education level, baseline (ref: no education)		
Primary school or below	1.18 (1.01, 1.39) *	0.85 (0.76, 0.96) *
Secondary school or above	0.93 (0.77, 1.13)	0.70 (0.60, 0.82) ***
Years lived in Hong Kong, per SD, baseline	1.10 (1.04, 1.16) **	0.90 (0.86, 0.94) ***
Alcohol drinking, prior (ref: none)	0.96 (0.82, 1.13)	1.05 (0.91, 1.21)
Smoking, prior (ref: none)	1.77 (1.46, 2.14) ***	1.01 (0.81, 1.25)
Number of chronic diseases, prior (ref: 0)		
1 or 2	1.14 (0.95, 1.38)	1.23 (1.06, 1.44) *
≥ 3	1.55 (1.28, 1.88) ***	1.45 (1.24, 1.71) ***
Self-rated health, prior		
Very poor, poor or fair	Ref	Ref
Good or very good	0.86 (0.77, 0.95) *	0.98 (0.90, 1.08)
Live alone, prior (ref: no)	1.02 (0.86, 1.21)	1.03 (0.89, 1.18)
Depression, prior (ref: no)	1.03 (0.87, 1.23)	0.82 (0.69, 0.96) *
MMSE, per SD, prior	0.81 (0.76, 0.86) ***	1.04 (0.98, 1.10)
CSI-D, per SD, baseline	0.83 (0.78, 0.89) ***	0.85 (0.80, 0.90) ***
Total PA, per SD, baseline	0.78 (0.74, 0.83) ***	0.88 (0.84, 0.92) ***
Built environment class, baseline (ref: Class 1)		
Class 2	1.07 (0.92, 1.24)	0.93 (0.83, 1.05)
Class 3	1.08 (0.92, 1.29)	0.98 (0.86, 1.12)

Abbreviations: CSI-D, Community Screening Interview for Dementia; MMSE, Mini-Mental State Examination; PA, physical activity; SD, standard deviation.

[a] Adjusted HR (95% CI) were estimated by generalized estimating equation with death (yes / no) and loss to follow-up due to other causes (yes / no) as outcomes. "Prior" means the predictors were treated as time-varying covariates, and "baseline" as baseline time-constant covariates.

* *P*-value<0.05, ** *P*-value <0.01, *** *P*-value <0.001

Table S3. Characteristics of participants and the level of physical activity over the study period

	2001-2003 (N=3944)	2005-2007 (N=3106)	2008-2010 (N=1848)	2015-2017 (N=1023)
	Mean (SD), median (IQR) or number (%)			
Age, years	72.5 (5.19)	75.7 (4.92)	77 (4.35)	83.3 (3.86)
Sex, female, N (%)	1972 (50.0)	1562 (50.3)	871 (47.1)	532 (52)
Marital status, N (%)				
Married	2795 (70.9)	2078 (66.9)	1291 (69.9)	589 (57.6)
Widowed	971 (24.6)	889 (28.6)	466 (25.2)	400 (39.0)
Separated or divorced	88 (2.2)	81 (2.6)	65 (3.5)	20 (2.0)
Single (never married)	90 (2.3)	58 (1.9)	26 (1.4)	14 (1.4)
Education level, N (%)				
No education	843 (21.4)	626 (20.2)	292 (15.8)	172 (16.8)
Primary school or below	1977 (50.1)	1555 (50.0)	918 (49.7)	483 (47.2)
Secondary school or above	1124 (28.5)	925 (29.8)	638 (34.5)	368 (36.0)
Years lived in Hong Kong	52.8 (14.9)			
Alcohol drinking, N (%)	515 (13.1)	356 (11.5)	227 (12.3)	94 (9.2)
Smoking, N (%)	273 (6.9)	171 (5.5)	67 (3.6)	25 (2.4)
Number of chronic diseases, N (%)				
0	648 (16.4)	277 (8.9)	154 (8.3)	41 (4.0)
1 or 2	2182 (55.3)	1422 (45.8)	780 (42.2)	435 (42.5)
≥ 3	1114 (28.3)	1407 (45.3)	914 (49.5)	547 (53.5)
Self-rated health, N (%)				
Very poor, poor or fair	2082 (52.8)	1545 (49.7)	909 (49.2)	559 (54.6)
Good or very good	1862 (47.2)	1561 (50.3)	939 (50.8)	464 (45.4)
Live alone, N (%)	423 (10.7)	437 (14.1)	244 (13.2)	170 (16.6)
Depression, N (%)	365 (9.3)	207 (6.7)	76 (4.1)	148 (14.5)
MMSE	25.6 (3.68)	26.1 (3.64)	26.9 (3.11)	24.8 (4.28)
CSI-D	30.2 (2.04)			
Insufficient PA, N (%), Total PA < 90	2223 (56.4)	1316 (42.4)	733 (39.7)	738 (72.1)
PASE, median (IQR)				
Total PA	84.8 (50.5)	95.6 (54)	99.1 (50)	68.4 (50.57)
Leisure PA	35.6 (34.5)	51.7 (35.6)	45.4 (29.6)	28.2 (37.08)
Household PA	50.0 (25.0)	50.0 (25.0)	50.0 (30.0)	25.0 (25.0)
Walking PA	25.7 (36.4)	25.7 (25.7)	25.7 (25.7)	25.7 (17.13)

Abbreviations: CSI-D, Community Screening Interview for Dementia; IQR, interquartile range; MMSE, Mini-Mental State Examination; PASE, Physical Activity Scale for the Elderly; SD, standard deviation.

Table S4. Sensitivity analyses for difference in the level of physical activity (PA) change over 5 years with built environment class [a]

	Model 3 (case = 3944; observation = 9921)	Model 3a (case = 3944; observation = 9921)	Model 3b (case = 3737; observation = 9140)	Model 3c (case = 2948; observation = 8696	Model 3d (case = 3944; observation = 8898)	Model 3e (case = 2928; observation = 7649)
Total PA						
Class 2 vs 1	0.16 (-1.66, 1.99)	0.19 (-1.62, 2.00)	0.53 (-1.37, 2.43)	-0.14 (-2.14, 1.87)	0.24 (-1.93, 2.41)	-0.54 (-2.71, 1.63)
Class 3 vs 1	-0.17 (-2.27, 1.92)	-0.09 (-2.16, 1.99)	-0.03 (-2.19, 2.13)	-0.45 (-2.76, 1.85)	0.06 (-2.42, 2.53)	-0.21 (-2.69, 2.28)
Class 3 vs 2	-0.34 (-1.93, 1.25)	-0.28 (-1.85, 1.30)	-0.56 (-2.19, 1.06)	-0.32 (-2.07, 1.43)	-0.18 (-2.05, 1.68)	0.33 (-1.59, 2.26)
Leisure PA						
Class 2 vs 1	-0.26 (-1.43, 0.91)	-0.21 (-1.36, 0.94)	-0.02 (-1.25, 1.22)	-0.34 (-1.62, 0.94)	0.40 (-0.91, 1.71)	-0.63 (-2.00, 0.75)
Class 3 vs 1	0.68 (-0.66, 2.03)	0.69 (-0.63, 2.01)	0.85 (-0.55, 2.25)	0.60 (-0.87, 2.07)	1.28 (-0.22, 2.77)	0.56 (-1.02, 2.13)
Class 3 vs 2	0.94 (-0.08, 1.97)	0.90 (-0.11, 1.90)	0.87 (-0.19, 1.92)	0.94 (-0.18, 2.06)	0.88 (-0.25, 2.00)	1.18 (-0.03, 2.40)
Household PA						
Class 2 vs 1	0.44 (-0.64, 1.51)	0.41 (-0.65, 1.47)	0.54 (-0.58, 1.66)	0.27 (-0.90, 1.45)	-0.22 (-1.48, 1.05)	0.29 (-0.98, 1.57)
Class 3 vs 1	-0.83 (-2.06, 0.40)	-0.78 (-1.99, 0.44)	-0.77 (-2.04, 0.51)	-0.97 (-2.32, 0.39)	-1.25 (-2.70, 0.19)	-0.62 (-2.09, 0.84)
Class 3 vs 2	-1.26 (-2.20, -0.33) **	-1.19 (-2.11, -0.27) *	-1.31 (-2.27, -0.35) **	-1.24 (-2.27, -0.21) *	-1.04 (-2.13, 0.05)	-0.92 (-2.05, 0.22)
Walking PA						
Class 2 vs 1	-0.46 (-1.33, 0.42)	-0.39 (-1.25, 0.47)	-0.27 (-1.19, 0.65)	-0.54 (-1.49, 0.40)	0.12 (-0.81, 1.06)	-0.67 (-1.69, 0.35)
Class 3 vs 1	0.73 (-0.27, 1.73)	0.77 (-0.21, 1.75)	0.95 (-0.10, 2.00)	0.67 (-0.42, 1.76)	1.18 (0.12, 2.25) *	0.73 (-0.44, 1.90)
Class 3 vs 2	1.19 (0.42, 1.95) **	1.16 (0.42, 1.90) **	1.22 (0.44, 2.01) **	1.21 (0.38, 2.04) **	1.06 (0.26, 1.85) **	1.39 (0.49, 2.30) **

[a] β (95% confidence interval) were estimated from weighted linear mixed-effects models.
Model 3: including age, built environment class and their interaction term (age*built environment class), and adjusted for sex, marital status, education level, alcohol drinking, smoking, years lived in Hong Kong, living alone, self-rated health, depression, Mini-Mental State, Examination Community Screening Interview for Dementia, number of chronic diseases and level of baseline total, leisure, household and walking physical activity, respectively.
Model 3a: repeated analyses of model using unweighted linear mixed-effects models;
Model 3b: repeated analyses of model after excluding participants who reported moving from the baseline address during follow-up;
Model 3c: repeated analyses of model after excluding participants who loss to follow-up within 4 years after baseline;
Model 3d: repeated analyses of model after excluding observation in 2015-2017 follow-up;
Model 3e: repeated analyses of model after excluding participants who reported a functional impairment.
* *P*-value<0.05, ** *P*-value <0.01, *** *P*-value <0.001

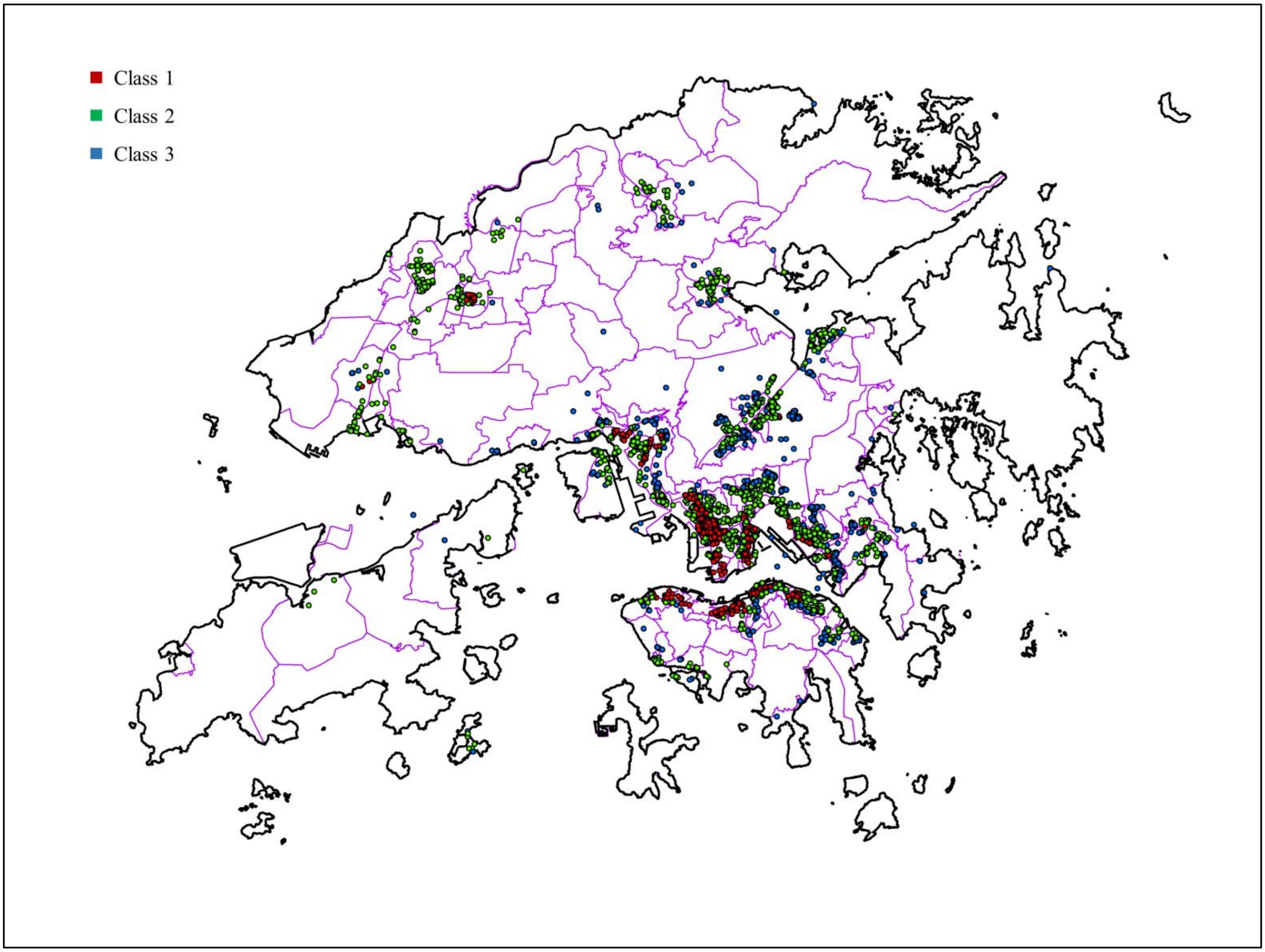

Figure S1. Geographical distribution of the participants' addresses at baseline.
